# Evaluation of nematode susceptibility and resistance to anthelmintic drugs with a WMicrotracker motility assay

**DOI:** 10.1038/s41598-025-02866-3

**Published:** 2025-05-23

**Authors:** Mélanie Alberich, Marie Garcia, Julie Petermann, Clara Blancfuney, Sophie Jouffroy, Philippe Jacquiet, Anne Lespine

**Affiliations:** 1 INTHERES, Université de Toulouse, INRAE, ENVT, Toulouse, France; 2 IHAP, Université de Toulouse, INRAE, ENVT, Toulouse, France

**Keywords:** Macrocyclic lactones, Motility, *Caenorhabditis elegans*, *Haemonchus contortus*, WMicrotracker, Microbiology, Experimental organisms, High-throughput screening

## Abstract

Grazing ruminants suffer from various helminth infections particularly those caused by gastrointestinal nematode (GIN) parasites, which have a considerable impact on their welfare and productivity. To treat these infections, the intensive use of macrocyclic lactone (ML) anthelmintics has led to the emergence of drug-resistant parasite populations worldwide. The standard method for detecting resistance, the Faecal Egg Count Reduction Test (FECRT), is susceptible to misinterpretation, leading to flawed management decisions that undermine parasite control efforts. Thus, there is a pressing need for robust resistance detection methods in field parasites. We investigated the potential of the WMicrotracker motility assay (WMA), previously unexplored in ML resistance assessment. The assay first compared ivermectin (IVM) susceptibility among wild-type Bristol N2 (N2B), IVM-selected (IVR10), and *nhr-8* loss-of-function (AE501; *nhr8(ok186)*) *Caenorhabditis elegans* strains. Dose-response curves indicated that IVR10 had a 2.12-fold reduction in sensitivity to IVM compared to N2B. Additionally, cross-resistance assessment showed that IVR10 exhibited decreased sensitivity to moxidectin (MOX) and eprinomectin (EPR) relative to N2B. Further investigation conducted on *Haemonchus contortus* revealed significant differences in drug potency between susceptible and resistant isolates, with MOX demonstrating the highest efficacy. Resistance factors (RF) highlighted the substantial resistance of the isolate collected in a farm with EPR-treatment failure. The WMA effectively discriminated susceptible from resistant isolates in both *C. elegans* and *H. contortus*. Our findings demonstrate, for the first time, the relevance of WMA as a phenotypic assay for detecting ML resistance in nematodes by measuring their motility response. This research sheds light on a novel approach for monitoring drug resistance, vital for effective parasite management strategies.

## Introduction

Parasitic nematodes pose a significant threat to animal health, with profound implications for livestock welfare and productivity. Among them, *Haemonchus contortus* stands out as a highly pathogenic nematode parasite, exacerbating the challenges faced by livestock owners and impacting overall animal well-being and economic output^1^. Today, the most reliable treatment for these diseases relies on the use of anthelmintic pharmaceuticals. Ivermectin (IVM) belonging to the macrocyclic lactone (ML) class, is one of the most important anthelmintic drug used worldwide in veterinary and human medicine^2^. Moxidectin (MOX), another ML was introduced to the veterinary market and has proven to be more effective than IVM against diverse resistant isolates of nematodes in different animal species^3^. In France, Eprinomectin (EPR) is the only anthelmintic drug that does not necessitate a milk withdrawal period during lactation, ensuring continued accessibility for dairy sheep, goats, and cattle throughout the lactating period. Inevitably, intensive use of an anthelmintic class has selected drug-resistant parasite populations globally in many animal species. This is now a major global problem in small ruminants, increasing in cattle^4^, and in some parasites of companion animals^5^. Nowadays, important losses in productivity in farm animals result from failure to control resistant worms adequately. The rapid spread of MLs resistance compromises not only the control of parasites in animals but also in humans^6,7^. Consequently, preventing, diagnosing, and managing anthelmintic resistance (AR) take precedence as primary research focuses in veterinary helminthology^8^ and is also a concern for control of nematode parasites in humans^9^.

In that context, actions are needed to detect drug resistance early, and to preserve efficacy of existing drugs as much as possible. This requires an in-depth understanding of the mechanisms of resistance to anthelmintics. Investigating mechanisms of AR in parasites is a challenging task because of their complex life cycle, relying on propagation in the host. Therefore, the free-living nematode *Caenorhabditis elegans* is a powerful and recognized model to study AR^10^. The use of this model system has considerably improved the understanding of the mechanism of action of anthelmintics, as the targets of some of them have been elucidated through advanced genetic screens for *C. elegans* mutants that were resistant to their effects^11^. *C. elegans* strains resistant to anthelmintics have evolved and are important models for understanding drug resistance^12–14^. Moreover, the approach of using *C. elegans* as an experimental model of parasitic nematodes is promising as it allows fast progress. Indeed, thanks to the use of this model, we have recently identified a new key regulator of IVM tolerance; the nuclear hormone receptor NHR-8^15^.

In the field, detecting resistance in gastrointestinal nematode parasites relies on the Faecal Egg Count Reduction Test (FECRT)^16^. This test compares egg counts prior to and post an anthelmintic treatment, computing a specific drug’s efficacy percentage. However, misunderstanding of potential factors that may affect FECRT results can prompt misguided decisions in management, with significant consequences for continuous parasite control^17^. In this context, there is an imperative to develop robust methods for detecting drug resistance in field parasites. Motility tracking system, proposed as a whole animal approach, has been initially developed to fully characterize the locomotor behavior and circadian rhythm of locomotor activity in adult *C. elegans*^18,19^. Subsequently, the usefulness of this test was quickly understood and multiple applications of the WMicroTracker One (WMi) emerged. Indeed, this assay has been used to study dye toxicity in *C. elegans*^20^. High throughput motility analysis has been reviewed for its possible application in the search for new anthelminthics in the context of resistance^21^. As examples, it has been used to screen anthelmintic activity of medicinal plants on *C. elegans*^22,23^ and of essential oil against *H. contortus*^24^. The interest concerning various parasites should be stated and a number of studies were carried out. It is in that context that WMi helped to demonstrate the promising anthelmintic properties of the repurposing drug EVP4593, as an anthelmintic on L_3_ of different parasite species such as *Cooperia oncophora*, *Ostertagia ostertagi*, *H. contortus* and *Teladorsagia circumincta*^25^. Then, the protocol to study worm motility with WMi has been improved as a high throughput test to study anthelmintic activity of large libraries of compounds on *H. contortus*^26,27^. In a recent study, Suarez et al.. investigated the interaction between IVM and EPR using WMi on *C. elegans*. Their findings dissuaded the concurrent usage of these drugs, a practice occasionally suggested by commercial formulations, as the combined effects were not superior to their individual actions^28^. In parallel, Automated Larval Migration Assay (ALMA) has also been adapted for high-throughput applications and has been proven effective for assessing *H. contortus* L_3_ motility, particularly in the determination of IC_50_ values for cholinergic agonists and MLs. While ALMA is based on migration analysis, WMi directly quantifies worm movement, providing a complementary approach that enables continuous, non-invasive motility assessment. The combination of these methods strengthens the toolkit available for large-scale drug screening and resistance monitoring in parasitology^29^. Nevertheless, given that the WMicrotracker motility assay (WMA) has not been previously applied in the context of ML resistance, the aim of this research was to evaluate its potential to discriminate susceptible from resistant nematodes. We aimed to assess computer-aided measurements of motility as a method for rapidly evaluating drug efficacy in nematodes and assessing their resistance status. Then, we have evaluated the suitability of such methods for assessing the drug tolerance to IVM, MOX and EPR of several *C. elegans* strains of known resistance status and tested its applicability on a field parasite of interest, *H. contortus*.

## Materials and methods

### Materials

IVM, MOX, EPR, dimethyl sulfoxide (DMSO), bacto agar, bacto peptone, bovine serum albumin (BSA), CaCl2, LB, NaCl and MgSO4 were purchased from Sigma-Aldrich (St Quentin Fallavier, France). For all experiments, IVM, MOX and EPR were dissolved in DMSO.

### *C. elegans* nematode strains and cultivation conditions

Wild-type *C. elegans* strain N2 (N2B) and the hypersusceptible mutant strain to IVM AE501 (*nhr-8*(ok186)), as well as the OP50 *Escherichia coli* strains were obtained from the *Caenorhabditis* Genetics Center (CGC, University of Minnesota, Minnesota, Minneapolis, MN, USA). The IVM-selected strain named IVR10, was kindly provided by Dr C. E. James^12^. All strains were cultured and handled according to the procedures described previously^13,15^. Briefly, nematodes were cultured at 21˚C on Nematode Growth Medium (NGM) agar plates (1.7% bacto agar, 0.2% bacto peptone, 50 mM NaCl, 5 mg/L cholesterol,1 mM CaCl2, 1 mM MgSO4, and 25 mM KPO4 Buffer) seeded with *Escherichia coli* strain OP50 as a food source. N2 Bristol and AE501 strains were cultured on classic NGM agar plate while IVM-selected strain (IVR10) was cultured on NGM plates containing 11.4 nM (10 ng/ml) of IVM. IVM-containing NGM plates were prepared as follows: stock solutions of IVM were diluted in NGM at the adequate concentration before pouring plates. Nematodes were synchronized through egg preparation with sodium hypochlorite. Briefly, an asynchronous population with majority of gravid adults and eggs was collected by washing the bottom of the NGM plates with M9 buffer (3 g KH2PO4, 6 g Na2HPO4, 5 g NaCl, 0.25 g MgSO4 7H2O in 1 l water) and centrifuged at 1300 g for 30 s. All worm stages except eggs were lysed with a bleaching mixture (5 M NaOH and 1% hypochlorite). Three washes of M9 were done to remove the toxic bleaching mixture. *C. elegans* eggs were then hatched overnight at 21˚C, on an orbital shaker, in M9 solution without bacteria to obtain a synchronized population of first-stage larvae (L_1_).

### *H. contortus* isolates

Two purified isolates of *Haemonchus contortus* were used in this study. The *H. contortus* isolate R-EPR1-2022 was originally obtained from a dairy sheep farm in South of France, recently diagnosed with clinic therapeutic failure by FECRT^30^, while S-H-2022 was a ML-susceptible isolate previously collected in a farm from the same region where EPR was still effective, and kept in the laboratory for several generations. The eggs were isolated from sheep feces collected either from a farm where EPR was effective, or from a farm where EPR resistance had been demonstrated. Since their recovery in 2020 and 2021, isolates were passaged twice per year in sheep infected with 10,000 infective larvae (L_3_) to maintain the strains. No ML drug pressure was applied during these passages. Experimentations involving sheep were performed in the experimental sheepfold facilities of the veterinary school of Toulouse ENVT (accreditation number E31 555 027). The project has been registered and authorized under the number APAFIS #40417–20230119164118 v3 by the French Ministry of Higher Education and Research.

### Worm motility assay (WMA)

**Fig. 1:**
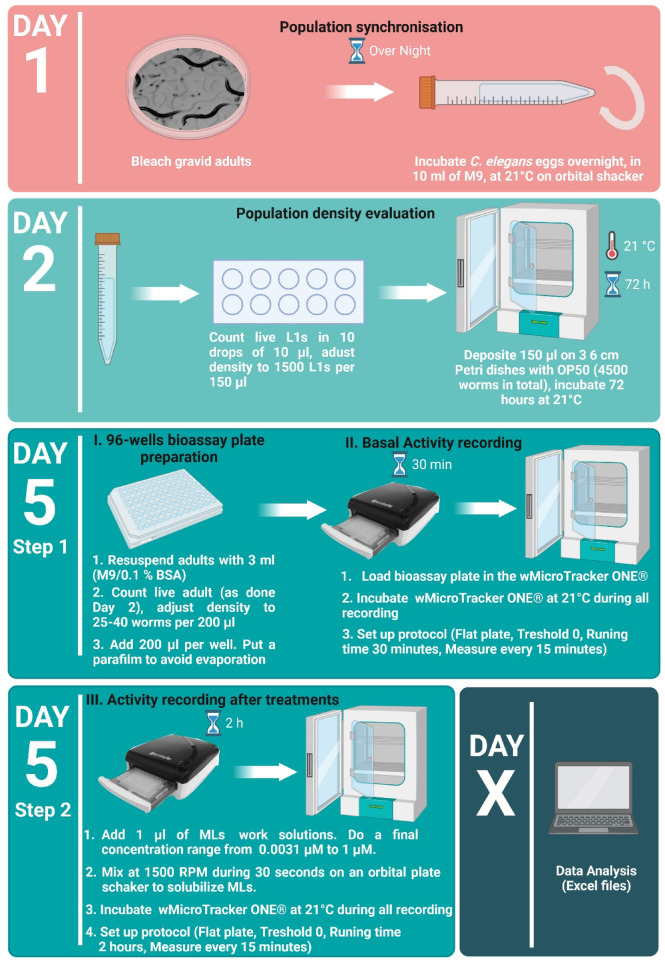
***Caenorhabditis elegans*** **experimental setup**. Schematic overview of the experimental procedure and associated timeline for culturing *C. elegans*. The timeline for preparing bioassay plate for worm motility measurement using WMi is also provided. To prevent adult sticking, all plastic material in contact with adult worm (Step 5) must be impregnated with 0.1% BSA (Bovine Serum Albumin). The figure is inspired by Figure from Preez et al., 2020^33^. Created with BioRender (https://www.biorender.com/).

**Fig. 2:**
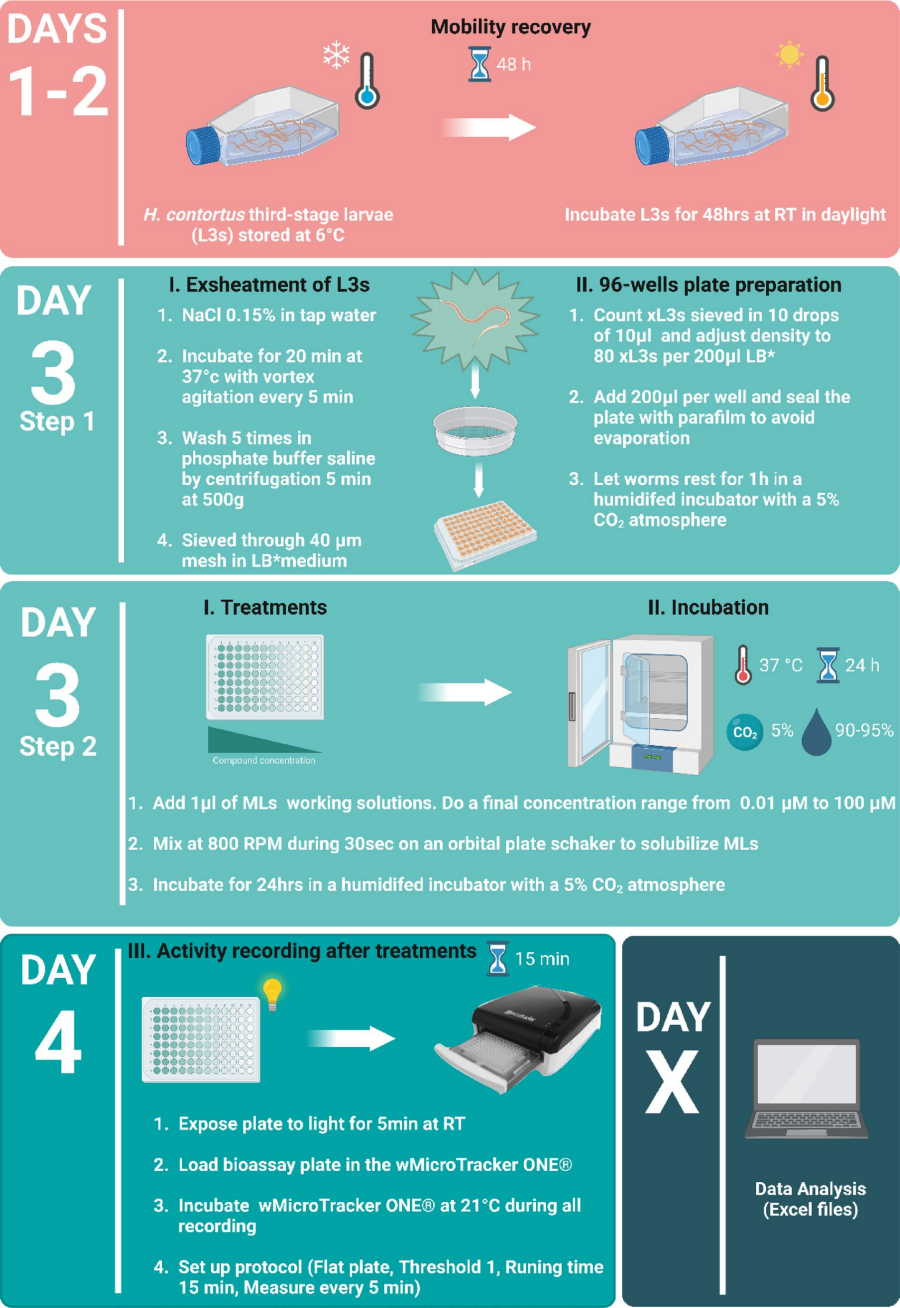
***Haemonchus contortus*** **experimental setup.** Schematic overview of the experimental procedure and associated timeline for or worm motility measurement using WMi. To prevent sticking, all plastic material in contact with the larvae must be impregnated with 0.1% BSA (Bovine Serum Albumin). LB*: Luria Bertani medium supplemented with final concentrations of 100IU/mL of penicillin, 100 µg/mL of streptomycin and 0.25 µg/mL of amphotericin B. The figure is inspired by Figure from Preez et al., 2020^33^. Created with BioRender (https://www.biorender.com/).

The susceptibility to MLs of the *C. elegans* strains and *H. contortus* isolates was determined in a WMA. Motility of nematodes was assessed from score activity recording using the WMicroTracker One (WMi) from PhylumTech (Santa Fe, Argentina), which detects infrared microbeam interruptions due to worm movement in liquid media. The method used has been adapted from protocols previously described for *C. elegans*^18–20,31^ and *H. contortus*^27^. The capacity of IVM, MOX and EPR to inhibit the worm motility was measured in a dose dependent assay by adding the drugs at increasing concentration (0.0031–1 µM for *C. elegans* and 0.01–100 µM for *H. contortus*). To prevent worm sticking and to ensure uniform distribution of the worms in each well, all plastic material must be coated with 0.1% BSA (Bovine Serum Albumin). The drugs were solubilized in DMSO, and the final concentration of DMSO in the assay was below 0.5%, to exclude harmful effects of the vehicle^32^. Given the significant procedural differences between *C. elegans* and *H. contortus*, in order to enhance clarity, a schematic overview of the WMi experimental procedure as well as the appropriate timeline inspired from Preez et al.^33^, is provided in Fig. 1 for *C. elegans* and in Fig. 2. for *H. contortus*.

#### WMA on *C. elegans* strains

The WMA on *C. elegans* measures the potency of anthelmintics in inhibiting motility in young adults. After bleaching, 4500 L_1_ were poured on 3 classic NGM agar plates. Synchronized young adults (40–50 per well) were seeded into a final volume of 200 µl M9 in a 96-flat well plate. Plates were incubated 25 min at 21 °C to allow the worms to settle. Then, basal activity was measured for 30 min to normalize the movement activity in each well at the beginning of the assay. Immediately after drug treatment, each score activity was recorded for a 120-minute period. Motility was calculated according to the formula:

(Score Activity _120 min_ Treated well – Score Activity _120 min_ Control DMSO without worms)

(Basal Activity _30 min_ – Basal Activity _30 min_ Control DMSO without worm).

Motility percentages were calculated for each treated well as –fold induction relative to DMSO treated worms which was set to 100. To facilitate comparison with other phenotypic assays, Table 1 provides a summary of IC_50_ values obtained from various assays in *C. elegans*.

**Table 1:** Comparison of macrocyclic lactones IC_50_s (nM) obtained by different *C. elegans* phenotypic assay.

**Assay**	**Informations about experiments**	***C. elegans*** **strains**										
		**N2B**			**NHR-8**				**IVR10**			
**Motility assay**		**IVM**	**MOX**	**EPR**	**IVM**	**MOX**	**EPR**		**IVM**	**MOX**	**EPR**	**References**
	Adults, Worm Microtracker	33.52	59.18	54.84	29.26	63.80	40.15		71.20	88.16	101.41	Current study
	L4, Worm Microtracker	190	*ND*	*ND*	*ND*	*ND*	*ND*		*ND*	*ND*	*ND*	^31^
	Adults, Worm Microtracker	290	120	*ND*	*ND*	*ND*	*ND*		*ND*	*ND*	*ND*	^34^
	L4, Worm Microtracker	150–500	*ND*	100–300	*ND*	*ND*	*ND*	*ND*	*ND*	*ND*	*ND*	^28^
**Larval development assay**	Agar plates	1.69	1.77	1.19	*ND*	*ND*	*ND*		12.43	3.06	17.82	^13^
	Agar plates	1.63	*ND*	*ND*	0.96	*ND*	*ND*		*ND*	*ND*	*ND*	^15^
**Pharynx pumping**	8-channel chip	1420	900	*ND*	*ND*	*ND*	*ND*		*ND*	*ND*	*ND*	^34^
	ScreenChip	51	42	*ND*	*ND*	*ND*	*ND*		*ND*	*ND*	*ND*	^34^

Mean IC_50_ (Inhibition Concentration for 50% inhibition) calculated by authors (See References)

ND (Not Determined)

#### WMA on *H. contortus* isolates

The WMA on *H. contortus* measures the potency of anthelmintics in inhibiting motility in exsheathed L_3_ larvae (xL_3_). Since xL_3_s have been shown to be at least 231 times more sensitive to MOX than L_3_s, demonstrating a significant increase in drug susceptibility without affecting larval viability, xL_3_s were used for WMA^27,34^. Then, prior to the experiment, *H. contortus* larvae were treated to discard cuticle. Briefly, worms were incubated 20 min at 37 °C, in tap water supplemented by NaCl 0.15% and vigorously shacken by vortex every 5 min. To prevent larval aggregation, larvae were filtered through a 40 μm mesh in LB medium. Each well of a 96-flat well plate received 80 xL_3_s suspended in a 200 µl final volume of LB medium. Then, the plates were treated with compounds and subsequently incubated at 37 °C for 24 h within a humidified incubator, maintaining a 5% CO_2_ atmosphere and humidity levels ≥ 90%. Following the incubation period, motility of the larvae (xL_3_) was restored by exposing them to light at room temperature for 5 min. Thereafter, the movement of worms within each well was recorded over a 15-minute duration using WMi technology.

#### Dose-response analysis and resistance factor (RF) calculation

The motility of worms in each well was then standardized against the average motility of control wells to derive the motility inhibition values (%). Post log_10_-transformation of compound concentrations, dose-response curves for the motility assay were fitted using a sigmoidal model with variable slope parameters. The 95% confidence limits were determined and graphed using GraphPad Prism (Version 6.01, https://www.graphpad.com). IC_50_ values, i.e., the concentration at which 50% of the worms are immobilized by the drug were then calculated (GraphPad, San Diego, CA, USA). Each value was the mean of triplicate and the experiments were performed at least 3 times. RF was the fold resistance relative to the susceptible worm population (N2B for *C. elegans* and S-H-2022 for *H. contortus*). It was equal to the IC_50_ for resistant population/IC_50_ for susceptible population.

### Statistical analysis

Firstly, the bioassay data were tested for normality using the D’Agostino & Pearson omnibus normality test. Thereafter, a two-way analysis of variance (ANOVA) was used to compare the effect of two categorical variables on the motility of worms: (i) biological condition (referring to drug tolerance status of strains; control, resistant or hypersensitive) and (ii) drug treatment condition (IVM, MOX and EPR) at different concentrations. Tuckey’s test was applied for multiple comparisons. Biological repeats are defined as independent experiments conducted on separate populations of animals on different days. These analyses were performed using GraphPad Prism6 software package (Version 6.01, https://www.graphpad.com).

## Results

### Motility assay to assess IVM efficacy in adult *C. elegans*

**Fig. 3:**
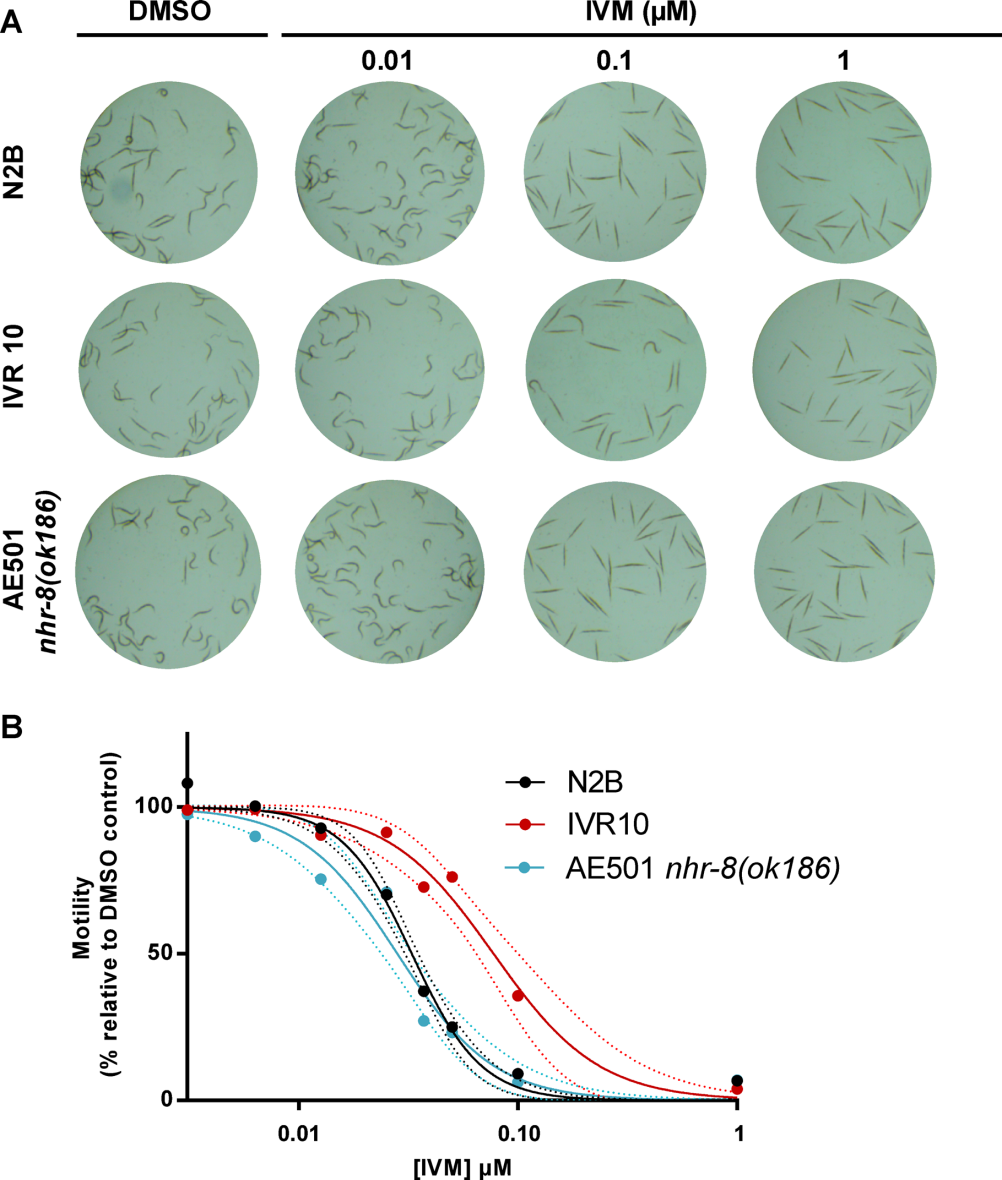
**Comparison of IVM efficacy in** ***C. elegans*** **strains using WMA. (A)** Top view of *C. elegans* adults in representative wells of 96-well plates after 120 min exposure without or with IVM at 0.01, 0.10 and 1.00 µM. **(B)** Representative concentration-response curve of *C. elegans* motility inhibition after exposure to IVM. Worm motility was assessed using the WMi which allows to quantify the locomotor activity of a worm population. Young adults of wild-type Bristol N2 (N2B, black), IVM resistant (IVR10, red) and IVM hypersensitive (AE501 *nhr8(ok186)*, blue) strains were incubated for 120 min at 21 °C in the presence of increasing concentrations of IVM (0.0125 to 1 µM). For each strain, motility percentages are expressed as –fold induction relative to DMSO treated worms, which is set to 100, and are reported as the mean and 95% confidence bands (dotted lines), a triplicates per conditions out of 5–20 experiments.

Different conditions were initially tested to obtain an optimal *C. elegans* motility response. We first verified that DMSO, used as a vehicle did not affect adult *C. elegans* motility when used at concentrations below 0.5%. Subsequently all experiments were conducted in medium with DMSO under 0.5%. We then determined the number of worms required for the experiment and established a linear correlation with motility between 30 and 90 worms (data not shown). The experiments were then performed with 40 worms per well, in the linearity range, and the motility was recorded during 120 min. We then used WMA to compare IVM susceptibility of several *C. elegans* strains: the wild-type Bristol N2 (N2B), IVM-selected (IVR10) and *nhr-8* loss-of-function (AE501; *nhr8(ok186)*) (Fig. 3).

Figure 3A shows representative images of worms after 120 min incubation without or with increasing concentration of IVM. Images clearly show nice curved animals in control (DMSO) or in IVM samples at 0.01 µM of IVM. In these conditions, animals were regularly moving revealing that such a low concentration of IVM did not affect worm motility whatever the *C. elegans* strain. IVM at 1 µM was able to induce total population body stiffness phenotype representing complete altered worm motility in all strains. Images reveal clear visible differences in motility phenotype between strains at 0.1 µM of IVM. All N2B and AE501 worms being immobile, while a significant proportion of IVR10 worms were still moving. To quantify such differences, dose-response curves for IVM toward motility of young adult *C. elegans* of the three strains were graphed and are presented in Fig. 3B. IC_50_s, i.e., the concentration of IVM at which 50% of animals are immobile and RF values, reflecting differences in the IC_50_ compared with that of N2B, are shown in Table 2.

**Table 2:** Effect of IVM, MOX and EPR on worm motility in wild-type N2B, IVM-selected (IVR10) and IVM-hypersensitive *nhr-8*-deficient (AE501 *nhr-8(ok186)*) *C. elegans* strains.

	N2B		IVR10		AE501 nhr-8(ok186)	
Treatment	Mean IC_50_ (nM) ± SD (no. of expts)		Mean IC_50_ (nM) ± SD (no. of expts)	RF	Mean IC_50_ (nM) ± SD (no. of expts)	RF
IVM	33.52 ± 8.89 (20)		71.20 ± 26.49 (12)^a^	2.12	29.26 ± 6.33 (5)^e^	0.87
MOX	59.18 ± 25.04 (11)^d^		88.16 ± 15.86 (4)^bd^	1.49	63.80 ± 21.35 (5)^df^	1.08
EPR	54.84 ± 16.37 (7)^d^		101.41 ± 2.09 (4)^ad^	1.85	40.15 ± 12.72 (5)^ce^	0.73

IC50 (Inhibition Concentration for 50% inhibition) calculated from motility assay.

RF is the fold Resistance relative to the N2B strain.

a P < 0.0001 versus N2B

b P< 0.01 versus N2B

c P< 0.5 versus NB2

d P< 0.0001 versus IVM treatment

e P< 0.0001 versus IVR10

f P< 0.01 versus IVR10

In agreement with the images, dose-response curves showed that IVM was able to alter worm motility of all strains. However, IVM displayed different potencies in affecting *C. elegans* motility. Indeed, similar potency of IVM to inhibit motility was observed for N2B and AE501 worms as shown by the superposition of the two dose-response curves and by the comparable IC_50_ values (33.52 ± 8.89 nM and 29.26 ± 6.33 nM, respectively). In contrast, the dose-response curve was significantly shifted to the right for the IVM-selected strain, reflecting a decrease in susceptibility to IVM. Indeed, IC_50_ of IVM was 2.12-fold higher for IVR10 than wild-type (71.20 ± 26.49 nM and 33.52 ± 8.89 nM respectively, *P* < 0.0001) in agreement with a higher tolerance of IVR10 worms to IVM when compared with wild-type N2B^13^.

Because cross-resistance between MLs is well described^13^, we then investigated the ability of the motility test to discriminate ML tolerance between strains, by studying MOX and EPR impact on the motility of each strain. Table 2 shows that IVR10 strain was more tolerant to the three drugs tested compared with the wild-type strain with IVM being the most potent drug. Moreover, IVR10 strain was 1.85-fold and 1.49-fold less sensitive to EPR and MOX respectively when compared with N2B strain. IVM was also the most potent drug for N2B strain (Table 2). The trend towards greater IVM efficacy on worm motility phenotype seems to be continuing also in AE501 worm, however, differences are not significant compared with EPR treatment. The least potent drug toward motility in this strain was MOX. Taken together, these results clearly show that WMA is suitable to evaluate ML toxicity and to discriminate drug resistant from susceptible *C. elegans* strains but not appropriate to discriminate hypersensitivity of AE501 worms.

### Motility assay to discriminate susceptible from resistant *H. contortus* in the field

**Fig. 4:**
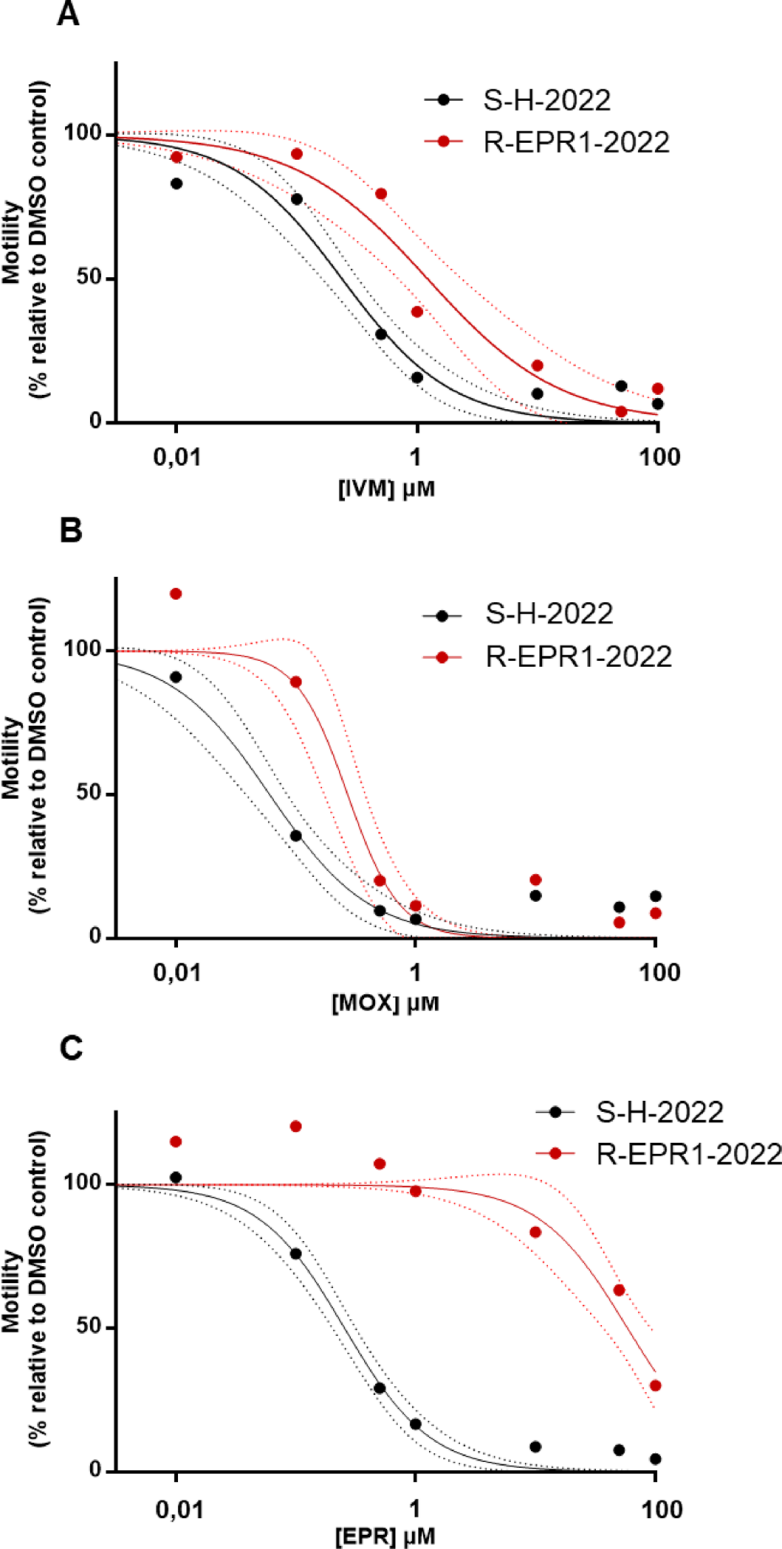
**Comparison of ML susceptibility of the ML-susceptible (S-H-2022) and ML-resistant (R-EPR1-2022)** ***H. contortus*** **isolates in a WMA.** Representative concentration-response curve of *H. contortus* motility inhibition following exposure to **(A)** IVM, **(B)** MOX and **(C)** EPR. Worm motility was assessed using the WMi which allows to quantify the locomotor activity of a worm population. xL_3_ of susceptible (S-H-2022, black), MLs resistant (R-EPR1-2022, red) strains were incubated for 24 h at 37 °C in a humidified incubator with a 5% CO2 atmosphere in the presence of increasing concentrations of IVM (0.01 to 100 µM). For each strain, motility percentages are expressed as –fold induction relative to DMSO, which is set to 100, and are reported as the mean and 95% confidence bands (dotted lines), a triplicates per conditions out of 3-7experiments

To respond to the urgent need of veterinarians and farmers to confirm resistance, in the context of treatment failure and suspicion of drug resistance in the field, we decided to adapt the use of WMi based on previous work^26^, to monitor motility in *H. contortus* and discriminate drug susceptible isolates from those developing drug tolerance. The number of *Haemonchus contortus* larvae per well was optimized by establishing a linear correlation between motility and worm count, confirming a linear range between 20 and 100 worms (data not shown). Based on this, experiments were conducted using 80 worms per well. The results of the dose-response experiments with IVM, MOX and EPR against an *H. contortus* isolate issued from a farm with therapeutic failure was compared with the ML-susceptible isolate in Fig. 4.

As expected, increasing concentrations of all drugs were able to alter worm motility of both strains. However, there was a significant difference on their potency. IC_50_ values for each drug on both isolates have been calculated and are presented in Table 3.

**Table 3:** Effect of IVM, MOX and EPR on worm motility in MLs-susceptible (S-H-2022) and MLs-resistant (R-EPR1-2022) *H. contortus* isolates.

	S-H-2022	*R*-EPR1-2022	
Treatment	Mean IC_50_ (µM) ± SD (no. of expts)	Mean IC_50_ (µM) ± SD (no. of expts)	RF
IVM	0.21 ± 0.12 (7)	1.10 ± 0.42 (3)^a^	5.24
MOX	0.05 ± 0.04 (6)	0.27 ± 0.06 (3)^bc^	5.40
EPR	0.26 ± 0.09 (7)	60.89 ± 16.92 (4)^ad^	234.19

IC_50_ (Inhibition Concentration for 50% inhibition) calculated from motility assay.

RF is the fold Resistance relative to S-H-2022.

^a^ *P* < 0.0001 versus S-H-2022

^b^ *P* < 0.001 versus S-H-2022

^c^ *P* < 0.05 versus IVM treatment

^d^ *P* < 0.0001 versus IVM treatment

Firstly, all three drugs were more effective on the reference strain than to the other field isolate, as demonstrated by the lower IC_50_ values, revealing that the worms collected form the farm that have no ML-susceptibility, were clearly ML-tolerant, when compared to the sensitive counterparts. Secondly, on both isolates, MOX was the most potent drug when compared with IVM and EPR as shown by the smaller IC_50_ within isolates (*P* < 0.0001 versus IVM treatment.). However, the potency of MOX and IVM was similarly low in the resistant isolate, revealing high tolerance of these worms to both drugs, as demonstrated by the same RF (5.24 and 5.40 for IVM and MOX, respectively, *P* < 0.0001 versus susceptible isolate). The most substantial degree of resistance of the resistant isolate was observed for EPR. Indeed, the RF was 234, considerably high for this substance and is reflected by a huge shift of the curve to the right. Indeed, EPR was the least potent compared with the two other substances, while IVM displayed intermediate potency.

## Discussion

To establish robust nematode control programs, it is essential to integrate highly sensitive and easy methods for detecting and regularly monitoring AR. However, in practical farm applications, the available assays are often laborious. Reduced motility is a key phenotype for evaluating the bioactivity of anthelmintic compounds. In this study, we sought to evaluate the feasibility of measuring the WMA using the automated apparatus WMi, as a reliable and relatively rapid method for assessing ML susceptibility in nematodes. Our final objective was to discriminate between susceptible and resistant nematodes. We demonstrated, for the first time, the reproducibility of this method, showcasing its sensitivity in differentiating between susceptible and resistant isolates of *C. elegans* and *H. contortus*.

The primary objective of our investigation was to conduct a comparative analysis of the efficacy of the anthelmintics IVM, MOX and EPR on both susceptible and resistant strains of the nematode model *C. elegans*, as well as the parasite of ruminants *H. contortus*.

As a preliminary step, we first explored the efficacy of WMA in distinguishing between susceptible and resistant *C. elegans* strains, using this readily available and easily maintained model—free from the constraints of infected host animals^11^—to validate and optimize the test before applying it to parasitic nematodes. Although FECRT remains the recommended test for assessing drug efficacy on farms, the LDA is one of the preferred methods for evaluating ML efficacy in *C. elegans* and gastrointestinal nematodes^35–38^. Subsequently, we explored the potential application of the WMA within the nematode model *C. elegans*. In this study, we conducted comparative analyses of the impact of MLs on worm motility inhibition across three strains: the wild-type Bristol N2 strain (N2B), the IVM-selected strain IVR10 and the IVM-hypersensitive AE501 strain. While the WMi assay has been previously employed to monitor ML efficacy in *C. elegans*^31^, this study marks the first instance where its utility was explored to compare drug resistant and susceptible strains. As expected, IVM, MOX and EPR were able to alter worm motility of all strains studied and differences in potency of the drugs were observed between strains. Indeed, IVM was able to alter N2B worm motility with very high potency, while MOX and EPR showed comparable potency, lower than IVM. We then measured the impact of ML on worm motility of ML-resistant *C. elegans*. This strain has been selected on IVM pressure and is highly resistant to the three MLs, as determined by LDA^13^. Significantly higher concentrations of the three MLs were required to affect the worm motility of the IVM-selected strains, showing that IVR10 strain was not only tolerant to IVM but also to MOX and to EPR, as consistently observed in the LDA data. Interestingly, our results indicated equal potency of IVM in inhibiting the motility of both the parental N2B strain and the IVM-hypersensitive strain, as evidenced by their identical RF, while by using LDA AE501 strain shows hyper-susceptibility to IVM compared with N2B^15^. These data indicate a potential limitation of sensitivity of the motility test compared with the LDA. Nonetheless, the WMi assay was shown to be effective in discriminating between N2B and IVR10. Comparative analyses of the three substances revealed IVM as the most potent drug in the N2B and IVR10 strains using WMi. This aligns with previous findings demonstrating the sensitivity of N2B motility to IVM and MOX^36^. However, while the trend towards greater IVM potency persisted in AE501 worm, differences were not significant compared with EPR treatment (Table 2). This reveals differences with previous LDA data, showing a higher potency of EPR in IVR10 worms compared with N2B. This is certainly due to the drug effect on the phenotype observed which is different from one test to another. Motility is affected by ML in a different way than larval development. This reveals discrepancies between tests and encourage one to take into account the assays accuracy when choosing a test to evaluate drug efficacy. MLs, and especially IVM, are well characterized for its inhibitory effect on worm motility. Nevertheless, disparities in concentration with other studies were observed (Table 1). Indeed, our results showed a significant motility inhibition at ML concentrations lower than those used in previous studies. As an example, Hahnel et al. conducted a comparative analysis of the potency of IVM and MOX in suppressing N2B motility by using WMi. Their findings revealed that MOX exhibited 2.4 times greater potency in inhibiting motility compared to IVM in their experiment^39^. In the same range, Risi et al. tested the effect of IVM on *C. elegans* L4 motility and obtained an IC_50_ value of 190 nM^31^. Such discrepancies could be attributed to the specific method employed for the application of ML on the worm, which account for drug bioavailability in the assay. In our approach, we directly introduce 1 µl of ML into the well without prior dilution in another container. The reduced bioavailability of ML may be associated with the retention of ML in plastic, and bioavailability is contingent on this factor. We recommend avoiding ML contact with multiple containers to improve ML availability during assay. Table 1 further highlights that the IC_50_ values are notably lower for LDA than those obtained from motility tests. It could be explained, as illustrated by Munguia et al., that the extended read-out times of the LDAs in *H. contortus* resulted in notably heightened sensitivity to standard anthelmintics, including IVM, compared to a 24-hour test directly measuring the effects of anthelmintics on L_3_ motility^27^.

Taken together, these results preliminarily demonstrate the utility of WMi in assessing ML response in *C. elegans*. However, while the LDA effectively discerns hypersensitivity AE501 worms, the WMi assay did not reveal such heightened sensitivity to the drug. Nevertheless, our study highlights the complexity of interpreting ML concentration responses across various studies, owing to variations in drug application methods, phenotypes assessed, and other contributing factors (time of incubation, *C. elegans* development stage…).

While the fecal egg count reduction test (FECRT) is presently the favored approach for identifying AR at the farm level^38,40^, it bears the limitations of being labor-intensive, costly, and capable of detecting resistance only when it has already reached relatively high levels. In vitro assays are regarded as the most efficient for the early detection of AR^41–43^. In this context, monitoring worm motility via the larval migration assay in *H. contortus* has been suggested^44,45^. However, the manual counting of larvae in each well, which is labor-intensive, could be one of the reasons why this test isn’t commonly employed in the field for detecting drug resistance. Therefore, we studied WMi application on *H. contortus* L_3_ larvae and compared ML efficacy in two isolates, one susceptible and one, collected in farm, suspected of being ML resistant. Indeed, the IC_50_ values obtained for IVM and MOX (Table 3) were consistent with the concentrations previously reported for motility assays in xL_3_s after 24 h of incubation^26,27^. Then, we observed lower efficacy for the three drugs in the resistant isolates. The RF, for the 3 MLs, between susceptible and resistant isolates of *H. contortus* was consistently greater than 5, indicating a significant level of resistance to these 3 drugs. MOX was more potent than IVM, while EPR was the least potent drug, displaying the highest RF compared to IVM and MOX (RF up to 234). However, IVM and MOX displayed the same RF (5.24 and 5.40 respectively). The observed order of potency of MLs for *H. contortus* R-EPR1-2022 closely mirrored that observed in the resistant *H. contortus* Kokstad isolate, highlighting MOX as significantly more potent than IVM and EPR^13,46^. To our knowledge, this is the first report showing the significance of high-throughput quantitative motility assessment using WMi in detecting AR in a field parasite. Studies have shown that simpler and faster diagnostic tools may support greater uptake in veterinary practice, although implementation can vary depending on context and perceived barriers^47,48^. Interestingly, in just a four-day period on the L_3_ stage and with minimum human intervention, our results showed first that we were able to evaluate and compare ML efficacy on susceptible *H. contortus* isolates. Furthermore, our findings suggest that the WMA, using microtracker technology, could provide an efficient and practical method for assisting veterinarians and farmers in the field in detecting AR in *H. contortus* isolates, pending further validation on real field samples. The ALMA method was initially developed to evaluate levamisole or pyrantel susceptibility modulation in *H. contortus* L2 larvae co-exposed to siRNA targeting *Hco-acr-8*^49^. It was later applied to susceptible (S, Weybridge) and resistant (R, Kokstad) *H. contortus* isolates, demonstrating its potential for systematic resistance determination and novel drug screening. However, to induce AR of the resistant isolates, they treated the sheep with the three anthelmintics (levamisole, pyrantel, and IVM) after infestation, resulting in an RF of 15.1 for IVM^29^. In contrast, using WMi, we found on our resistant isolates a RF of 5 reflecting the inherent resistance of our isolates, without the additional factor of inducing resistance through treatment. Indeed, it is crucial to note that our *H. contortus* isolates were not previously re-treated with drugs, after the EPR-failed treatment, and were naturally more tolerant to EPR, without IVM treatment. This further supports cross-resistance among MLs. Concerning WMi, Munguia et al. assessed drug susceptibility in *H. contortus* xL_3_ larvae, aligning with our results for susceptible isolates (Kirby), but lacking data on resistant strain. Their automated xL_3_ assay allowed for high-throughput compound screening but showed lower susceptibility, requiring higher anthelmintic concentrations than adult-stage assays^27^. Our study demonstrated that WMi effectively differentiates between susceptible and resistant *H. contortus* worms using purified field isolates that are naturally highly tolerant to EPR, without laboratory-induced resistance. This better reflects real-world conditions and highlights the adaptability of this method for assessing drug efficacy in field isolates, particularly in detecting emerging resistance. Furthermore, it should improve our understanding of how subpopulations within a larger population can influence overall resistance, which is essential for effectively monitoring and managing drug resistance dynamics. By directly quantifying motility, WMi provides a robust approach for evaluating AR across diverse *H. contortus* populations. Finally, few studies have explored the motility test as an effective method for detecting AR. As an example, a report suggested that motility of the L_3_ stage was a poor phenotype for detecting and measuring AR of different gastrointestinal nematodes^50^. Indeed, in this study, RF never achieved more than 2 in score whatever the parasite being tested. These differences may be explained by (i) the test used which was based on larval migration less sensitive than the motility assay used in the present study and (ii) the presence of the cuticle as authors worked on sheathed L_3_ which is a robust barrier, shielding the worm from its environment, especially against xenobiotics^51^. We conducted our research on xL_3_ larvae, which guarantee that both susceptible and resistant L_3_s were optimally exposed to the drug. Another work on the filarial nematode *Dirofilaria immitis*, has shown that motility of microfilaria was not a reliable phenotype for detecting resistance in this parasite, encouraging professional to in vivo assay such as the microfilaria suppression test in the presence of a suspect case of resistant isolates^52^. Additional investigations are needed to explore its potential broader applications to other parasites and anthelmintic substances. Indeed, the WMA is also highly appropriate for measuring the activity of other anthelmintics acting in the nematode’s neuromuscular system, in particular the agonists of nicotinic acetylcholine receptors (nAChRs) such as levamisole. By contrast, WMi is not the most direct approach for assessing activity of benzimidazoles on *H. contortus*, given their mode of action through B-tubulin. Alternative methods like egg hatch assays (EHA) or LDA are more appropriate. Additionally, pyrosequencing, PCR, and the Nemabiome approach are well-suited for detecting BZ resistance, providing faster results, particularly when applied directly to eggs^53^. Moreover, recent studies in Australia have highlighted the effectiveness of using L_3_ and the Nemabiome method for BZ detection^54^. It should be of great interest to further develop the BZ Nemabiome technique for eggs to achieve even faster results.

While we demonstrate that WMi is a relevant assay for assessing ML resistance in *H. contortus*, several key challenges remain. Initially, we focused on purified *H. contortus* to validate the method, as this species often dominates after anthelmintic treatment in warmer climates where it is prevalent. The WMi strategy appears particularly relevant for implementation in hot and humid climates, where *H. contortus* is highly prevalent and often the dominant parasite, as reported in regions such as Australia^55^. Although *H. contortus* typically coexists in temperate areas with other gastrointestinal nematode species, it frequently remains the most drug-resistant species^56^. These findings support the broader applicability of the WMi approach across diverse environmental and epidemiological contexts. However, field samples frequently contain a mix of nematode species, which poses challenges for the WMi technology. Addressing how species composition in field samples affects assay reliability will be a critical next step. Moreover, WMi needs to be adapted for other common nematode species, such as *Teladorsagia circumcincta*, which co-infect sheep and are indeed often the dominant species in colder climates.

Another limitation is the potential presence of mixed populations of resistant and sensitive nematodes within a single sample, which could hinder resistance detection. To overcome this, experiments titrating various ratios of resistant and sensitive nematodes should be conducted. Additionally, the availability of a reference for sensitive isolates is an important requirement. To establish robust reference values for drug potency, the number of assays needs to be expanded to generate reliable benchmarks for sensitive isolates for each nematode species. These efforts will help validate the method for field use and provide a foundation for future research aimed at improving ML resistance detection. The resistance to MLs in field populations, particularly in certain production areas such as Pyrénées-Atlantiques, where resistance to EPR is dangerously spreading, underscores the critical need for rapid and reliable methods to identify and differentiate between susceptible and resistant populations^30^. Such tools are essential for implementing appropriate countermeasures when resistance is present and for minimizing treatments—and thus selection pressure—when resistance is not yet established, thereby supporting the sustainable use of anthelmintics wherever possible. Another key approach is refugia-based control, which preserves untreated parasite populations to slow anthelmintic resistance. However, its effectiveness remains to be confirmed, requiring further research to refine its application^57^.

In conclusion, our findings highlight for the first time, that the WMA stands as a robust test to be employed for effectively discerning between ML-susceptible and resistant *C. elegans* and *H. contortus* nematodes. While the assay requires 4–5 days, it remains significantly faster than many currently available tests, particularly for MLs, which still lack a genetic marker for standalone molecular diagnostics. Given this advantage, our method represents a relatively high-throughput approach compared to traditional techniques, facilitating a more rapid and reliable differentiation between susceptible and resistant populations. Moreover, this test could be a valuable tool to detect drug resistance in *H. contortus* as it allows to (i) discriminate ML-resistant from susceptible isolates of *H. contortus* from the field, (ii) show cross-resistance to the three substances in resistant isolates, and (iii) highlight a huge resistance to EPR consistent with the failure of treatment reported from the field.

## Data Availability

All data are fully available without restriction. The data is available at: https://doi.org/10.57745/RTLJVH.
